# Variability and Number of Circulating *Complementary Sex Determiner* (*Csd*) Alleles in a Breeding Population of Italian Honeybees under Controlled Mating

**DOI:** 10.3390/genes15060652

**Published:** 2024-05-21

**Authors:** Maria Grazia De Iorio, Barbara Lazzari, Licia Colli, Giulio Pagnacco, Giulietta Minozzi

**Affiliations:** 1 Department of Veterinary Medicine and Animal Sciences, Università degli Studi di Milano, 26900 Lodi, Italy; giulietta.minozzi@unimi.it; 2 Institute of Agricultural Biology and Biotechnology, Consiglio Nazionale delle Ricerche, 20133 Milano, Italy; barbara.lazzari@gmail.com (B.L.); giulio.pagnacco@unimi.it (G.P.); 3 Department of Animal, Nutrition and Food Sciences and Research Center on Biodiversity and Ancient DNA, Università Cattolica del Sacro Cuore, 29122 Piacenza, Italy; licia.colli@unicatt.it

**Keywords:** *Apis mellifera*, breeding population, *complementary sex determiner* gene, hypervariable region, alleles variability

## Abstract

In *Apis mellifera*, *csd* is the primary gene involved in sex determination: haploid hemizygous eggs develop as drones, while females develop from eggs heterozygous for the *csd* gene. If diploid eggs are homozygous for the *csd* gene, diploid drones will develop, but will be eaten by worker bees before they are born. Therefore, high *csd* allelic diversity is a priority for colony survival and breeding. This study aims to investigate the variability of the hypervariable region (HVR) of the *csd* gene in bees sampled in an apiary under a selection scheme. To this end, an existing dataset of 100 whole-genome sequences was analyzed with a validated pipeline based on de novo assembly of sequences within the HVR region. In total, 102 allelic sequences were reconstructed and translated into amino acid sequences. Among these, 47 different alleles were identified, 44 of which had previously been observed, while 3 are novel alleles. The results show a high variability in the *csd* region in this breeding population of honeybees.

## 1. Introduction

The *complementary sex determiner* (*csd*) gene plays a primary role in the sexual regulation of honeybees [1]. Honeybees are haplodiploid, with females (queen and workers) being diploid and males (drones) being haploid [2]. Diploid workers and queens are heterozygous for *csd,* while haploid drones are hemizygous. In the case of *csd* homozygosity, diploid drones develop, but they are identified and eaten by worker bees during the larval stage [3].

The *csd* gene comprises nine exons, including the potential specifying domain region (PSD) located within exons six to nine. Within the PSD a highly polymorphic region defined as the hypervariable region (HVR) is present. The HVR region differs greatly between alleles due to the varying number of asparagine/tyrosine repeats [4]. Based on current knowledge, it has been demonstrated that in *Apis mellifera* five amino acid differences and length variations in the PSD region are sufficient to produce diploid females regularly [5].

The *csd* gene has evolved under a balancing selection that is strongly beneficial for heterozygotes, as homozygotes have zero fitness [6]. Consequently, new and rare alleles have an advantage because their probability of being homozygous is much lower than that of common alleles [1,6,7,8]. Furthermore, Lechner et al. showed that mutations in the HVR region occur 2.4 times more frequently than in microsatellites [9].

In nature, the combination of these selective forces, along with the polyandry of queens mating, help to maintain a large number of different circulating *csd* alleles [10]. The number of *csd* alleles was initially estimated to be about 10–13 [11], but further studies have identified a higher number of circulating alleles, ranging from 16 locally [12,13,14,15,16] to over 200 alleles worldwide [9,17,18,19]. In the Italian honeybee population, 88 different alleles have recently been identified by analyzing 125 colonies of different genetic types in 12 different Italian regions [20].

However, in a closed breeding population, some breeding practices, such as mating few selected breeders to produce numerous sister queens, could increase inbreeding level and consequently increase homozygosity. This could have significant implications for various productive and reproductive traits and in particular for the *csd* gene [21,22].

This article aims to investigate the diversity of *csd* alleles circulating in an Italian breeding population, where breeders are selected regardless of their *csd* alleles. For this purpose, we analyzed 100 colonies belonging to a breeding population, selected since 2015 for three different traits related to honey production, hygienic behavior, and docility behavior.

## 2. Materials and Methods

### 2.1. Data

One hundred worker colonies were sampled in June 2021 from a honeybee breeding farm located in Lombardy. One young worker bee was collected from each hive and stored at 4 °C in 1.5 mL Eppendorf tube filled with 99% ethanol.

The sampled colonies belong to a breeding population which undergoes a selection scheme in which selected virgin queens are mated with selected drones using isolated mating stations. At the beginning of the selection project in 2015, tests were conducted to identify an area (valley) isolated from the presence of other drones. For this purpose, groups of virgin queens were brought to the site, and after a few weeks, it was verified that none of them had been fertilized/mated. Subsequently, the isolated mating station was protected by specific local ordinances, preventing the presence or introduction of other colonies unrelated to the project.

Each year, 108 colonies are phenotyped to estimate their breeding value (EBV) for honey production, hygienic behavior, and docility.

At the end of the season, the 7–8 colonies with the best EBV are selected as breeders for the next generation to produce the new 108 colonies. In detail, from the best colony, a group of 12 sisters is obtained through grafting, serving as drone-producing queens. In beekeeping, the drone-producing queens are considered as fathers since drones are haploid and descend from the queen; they can therefore be considered as spermatozoa of the queens. Simultaneously, the remaining top 6–7 colonies undergo grafting to produce 18 virgin queens each. Finally, the 108 virgin queens are mated in the isolated mating stations with the drone-producing queens to produce the new 108 colonies.

In order to ensure genetic diversity and avoid breeding from closely related colonies, the selection process alternates between two distinct groups of 108 colonies each year. This means that if one group is phenotyped and used for breeding in a particular year, the next year the other group is phenotyped and used for breeding. This ensures that the two groups do not share common ancestors in consecutive years.

**Figure 1:**
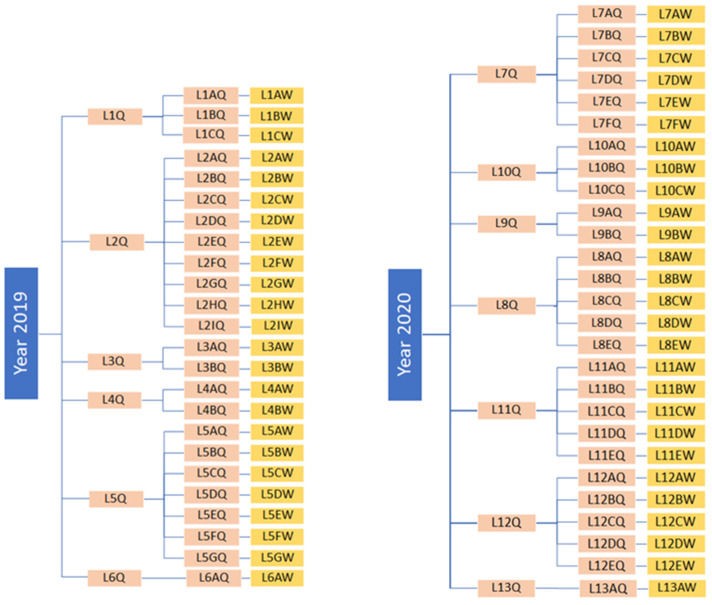
Genealogical trees illustrating the ancestry of the 51 analyzed worker bees. Queens are shown in pink and colonies are shown in yellow.

The 100 worker bees analyzed in this study belong to 100 different colonies founded in two different years and descend from 13 maternal lines. In detail, 40 colonies were driven by 40 queens born in 2019 from 6 selected breeding queens (group L1Q to L6Q), while the other 60 colonies were born in 2020 from 60 queens, daughters of 7 different queens (groups L7Q to L13Q). Therefore, these 100 colonies can be traced as descendants from 13 maternal lines. As the selection process involves the alternating use of 2 different groups, these 13 genetic lines of 2 consecutive years have no shared common relatives. More detail on the ancestry of the 51 worker bees for which HVR regions were reconstructed are shown in Figure 1. Moreover, Table 1 shows the distribution of the 51 samples among the 13 genetic lines of descent.

### 2.2. DNA Extraction, Library Preparation, Sequence Processing and Alignment

The whole insect was ground with liquid nitrogen and DNA was extracted using E.Z.N.A.^®^ Insect DNA Kit (Omega Biotek, Norcross, GA, USA) with one minor modification: the incubation with CTL Buffer and Proteinase K Solution was performed for 60 min instead of 30 min.

Celero™ DNA-Seq kit (Tecan Trading AG, Männedorf, Switzerland) was used for library preparation following the manufacturer’s instructions. Libraries were quantified by Qubit 2.0 and quality tested by Agilent 2100 Bioanalyzer High Sensitivity DNA assay (Agilent, Santa Clara, CA, USA). Libraries were then prepared for sequencing and sequenced with Illumina technology on NovaSeq 6000 in paired-end 150 mode. Reads were mapped to the reference genome (Amel_HAv3.1: GCF_003254395.2). Aligned sequences are only those that map to unique positions; duplicated sequences were marked by picard (https://broadinstitute.github.io/picard/, accessed on 1 February 2023) and removed from downstream analysis.

### 2.3. De Novo Assembly and Analysis of Sample-Specific HVR Allele Consensus Sequences

For each sample, Samtools was used to extract reads that mapped to exon 7 of the *csd* gene, which includes the HVR region [4]. The trimmed reads that passed quality control filters, were mapped to the Amel_Hav3.1 honeybee genome, resulting in a total of 7350 mapped reads corresponding to the HVR region (Hav3.1_CM009933.2:11771976-11772119). The CAP3 Sequence Assembly Program (-o 40, -p 90) [23], was used to cluster and generate the contigs. CAP3 output was qualitatively filtered. First, only samples with 2 contigs were retained, resulting in 70 samples. Then, an additional 19 samples with more than 1 singleton were excluded, leaving 51 samples that passed these 2 filtering steps. The two contig sequences obtained from each sample, supposed to represent the two alleles, were translated into amino acid sequences using EMBOSS transeq tool (https://www.ebi.ac.uk/Tools/st/emboss_transeq/, accessed on 10 March 2023). In-frame translations resulting in truncated polypeptides were discarded. The successfully reconstructed amino acid sequences numbered 102 and were aligned using Clustal Omega (1.2.2) [24].

## 3. Results

### Csd HVR Amino Acid Sequence

**Figure 2:**
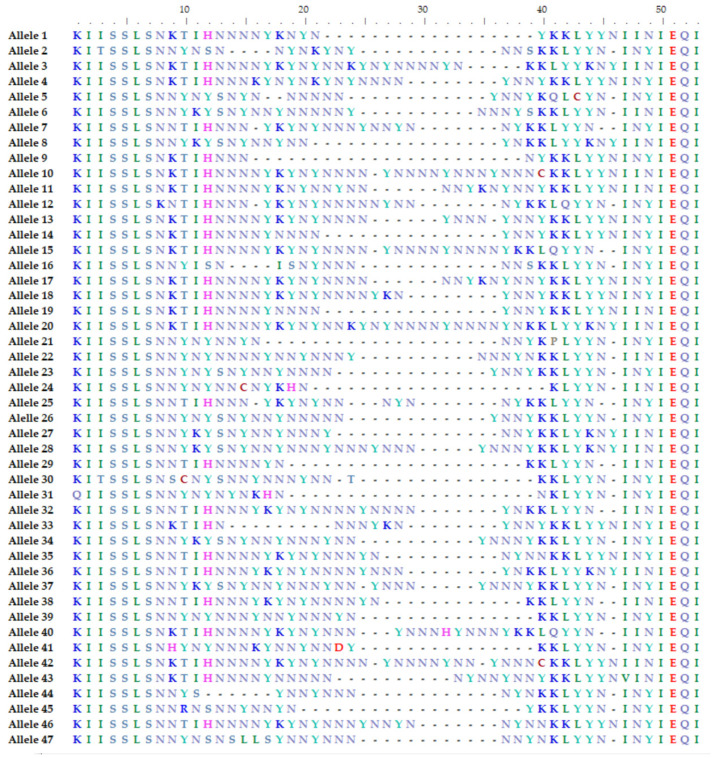
Alignment of amino acid sequences in the hypervariable region (HVR) of the *csd* alleles.

The hypervariable region (HVR) of the *csd* gene of 100 diploid worker bees belonging to 100 different colonies was sequenced using the pipeline described above. After de novo assembly and quality control filter, 102 amino acid sequences were successfully reconstructed. The obtained amino acid sequences were compared with a recent review that grouped 652 amino acid sequences into 225 alleles based on identity [19]. Each sequence was assigned an Amelcsd-HVR ID, numbered sequentially from the most to the least common (from Amelcsd-HVR1 to Amelcsd-HVR225) [19]. Out of the 47 alleles found, 42 were reported and named by Bilodeau et al., 2 alleles (allele 7 and allele 14) were described only by Paolillo et al. [20], while 3 (Allele 35, 39, 42) were novel alleles. In Supplementary Table S1, allele sequences are reported together with their frequencies and the corresponding Amelcsd-HVR numbers or Paolillo Allele numbers [19,20]. Moreover, Figure 2 visually represents the aligned amino acid sequences of the 47 alleles, highlighting the notable differences in both amino acid composition and length.

Furthermore, in line with the findings of previous studies, all the analyzed worker bees demonstrate heterozygosity and they exhibit notable differences in both length and sequence of the hypervariable region of the *csd* gene between the two carried alleles, as shown in Table 2.

**Figure 3:**
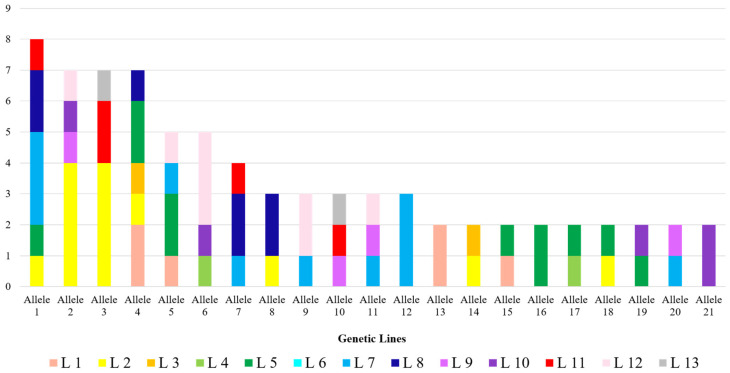
Histogram reporting the frequency of the 21 alleles found in at least 2 samples in the 13 genetic lines, the colors are given according to the genetic lines.

In total, 47 different sequences were found: 21 of these are shared in at least 2 samples and 26 are reported only once. Allele frequencies were determined by dividing the number of observed sequences by the total number of reconstructed sequences (102) and are reported in Table S1. The most frequent allele is Amel*csd*-HVR115 (allele 1), which is present in eight samples (frequency: 8%) belonging to five different genetic lines (two born in 2019 and three in 2020). In addition, the frequency of the 21 different alleles shared in at least 2 sample within the 13 genetic lines was analyzed. As described in the histogram reported in Figure 3, 17 out of 21 alleles were identified in at least 2 different genetic lines and, among these, 2 alleles (1 and 4) were reported in 5 different genetic lines.

## 4. Discussion

Some selection practices applied in bee breeding can increase inbreeding within colonies, resulting in increased homozygosity, especially if single drone insemination is used. The consequent reduced variability is problematic for productive and reproductive traits, particularly at the *csd* gene, causing colony losses [21,22]. Therefore, understanding the distribution and frequency of different *csd* alleles in a population of honeybees is important for beekeepers to make informed decisions about breeding practices and to maintain genetic diversity within their colonies.

This study aims to examine the effect of modern selection practices on the variation of the *csd* gene in a closed breeding honeybee population, using existing next-generation resequencing data to reconstruct *csd* alleles from diploid honeybees.

A bioinformatic pipeline was used for this purpose, which included manual review to achieve a curated, reference-independent sequence assembly. The pipeline was tested and validated by GenBank data in a previous study [20].

The HVR region sequence of 51% of the total samples in the study was successfully reconstructed. The obtained sequences were validated by comparison with known alleles found in the literature [19,20]. As shown in Table S1, 44 out of 47 alleles showed 100% identity with already-published sequences, while the remaining 3 are novel alleles. The results of this study confirmed that the *csd* gene is highly variable in both amino acid sequence and length. Moreover, the length of the alleles varies considerably, ranging from 30 to 53 amino acid sequences, which is in line with previous studies [15,19,20]. Table 2 further supports these findings, providing additional evidence of the extensive variability observed in the HVR region of the *csd* gene between the two alleles carried by the worker bees. This variability is consistent with previous studies [15,20].

The 51 colonies sequenced in the study belong to 2 generations and 13 genetic lines/breeding queens. Specifically, 24 colonies were born in 2019 and descended from 6 breeding queens, while the remaining 27 were born in 2020 and descended from 7 queens.

As shown in Figure 3, the different HVR sequences are well distributed among the genetic lines. This result indicates that there is still high variability within each line despite 6 years of selection.

In the present study, 47 different *csd* alleles are identified in a dataset of 102 sequences from 51 honeybees belonging to a breeding population. Paolillo et al. [20] found 88 alleles using the same pipeline on a dataset of 138 sequences from honeybees sampled from a variety of beekeepers across the Italian peninsula. Together, these results are in line with the findings of Lechner et al. [9], who identified 53 alleles locally and 87 in a larger dataset covering a broader geographic area.

Since homozygous individuals do not survive in a honeybee colony, the observed heterozygosity must be 1 by definition. Nevertheless, based on the allele frequencies detected in this paper (Table S1) an index of expected gene diversity at this locus can be computed as described by Nei [25]:

$$He=1-\sum_{i=1}^{k}p_{i}^{2}$$

In the present population, where a selection scheme has been in place since 2014, this index resulted in 0.96.

This result can be compared with findings from a closed breeding population screened for the *csd* genes in New Zealand [14]. In the study, the alleles of 42 queens were analyzed by sampling 6 drone pupae for each queen. They successfully obtained both allele sequences from 35 out of the 42 queens, while only 1 allele sequence was obtained from the remaining 7, resulting in a total of 77 amino acid sequences, identifying 16 different alleles [14]. Based on the frequencies provided in our population in this paper, we estimated that the expected gene diversity in this population was 0.92 in 2010 and 0.90 in 2011. Interestingly, in a previous investigation [20] based on a larger population (across Italian peninsula), the expected gene diversity index as 0.98 using the allele frequencies provided by the authors. Therefore, all these results show that despite selection, the apparent loss of heterozygosity in the csd allele remains relatively limited within this breeding population. Consequently, we think that the major concern about the risk that selection in honeybees can excessively erode the genetic diversity in this species, is largely overrated.

## 5. Conclusions

The results of this study provide a valuable insight into the variability of the HVR region in closed honeybee populations, and the potential impact of controlled mating practices on their genetic diversity. The study found a total of 47 different *csd* alleles from 51 diploid worker honeybees collected from a selected breeding population. The high number of alleles found agrees with previous studies on *csd* gene variability in honeybees, even if the bees in this study belong to a highly selected population. However, this study highlights the importance of considering the *csd* alleles of reproducers in beekeeping practices to maintain a healthy and diverse genetic pool. The pipeline used in this study can also be applied to evaluate other selective traits, providing important information for breeding programs aimed at enhancing honeybee colony health, production, and survival.

## Figures and Tables

**Table 1:** Number of analyzed samples for each year and Genetic line (GL).

Year	GL	N°
2019	L1Q	3
	L2Q	9
	L3Q	2
	L4Q	2
	L5Q	7
	L6Q	1
2020	L7Q	6
	L8Q	5
	L9Q	2
	L10Q	3
	L11Q	5
	L12Q	5
	L13Q	1

**Table 2:** Amino acid sequences from the hypervariable region of the *csd* gene of the 51 diploid worker bees (ID sample), their respective mother ID, and the genetic lines (GL).

ID Sample	Mother	GL	Sequences	ID Allele
L1AW	L1AQ	L1Q	KIISSLSNNYNYSNYNNNNNNYNNYKQLCYNINYIEQI	Allele 5
			KIISSLSNKTIHNNNNYKYNYNNNNYNNNYNNYKKLYYNINYIEQI	Allele 13
L1BW	L1BQ	L1Q	KIISSLSNKTIHNNNNYKYNYNNNNYNNNYNNYKKLYYNINYIEQI	Allele 13
			KIISSLSNKTIHNNNKYNYNKYNYNNNNYNNYKKLYYNINYIEQI	Allele 4
L1CW	L1CQ	L1Q	KIISSLSNKTIHNNNKYNYNKYNYNNNNYNNYKKLYYNINYIEQI	Allele 4
			KIISSLSNKTIHNNNNYKYNYNNNNYNNNNYNNNNYKKLQYYNINYIEQI	Allele 15
L2AW	L2AQ	L2Q	KITSSLSNNYNSNNYNKYNYNNSKKLYYNINYIEQI	Allele 2
			KIISSLSNKTIHNNNNYKYNYNNKYNYNNNNYNKKLYYKNYIINIEQI	Allele 3
L2BW	L2BQ	L2Q	KIISSLSNNYKYSNYNNYNNNYNNNYNNNYNNNYKKLYKNYIINIEQI	Allele 28
			KIISSLSNKTIHNNNNYKYNYNNNNYKNYNNYKKLYYNINYIEQI	Allele 18
L2CW	L2CQ	L2Q	KIISSLSNKTIHNNNNYKNYNNYKKLYYNINYIEQI	Allele 33
			KIISSLSNNYKYSNYNNYNNYNKKLYYKNYIINIEQI	Allele 8
L2DW	L2DQ	L2Q	KIISSLSNKTIHNNNNYKYNYNNKYNYNNNNYNKKLYYKNYIINIEQI	Allele 3
			KIISSLSNNYNYSNYNNYNNNNNYNNYKKLYYNINYIEQI	Allele 26
L2EW	L2EQ	L2Q	QIISSLSNNYNYNYNKHNNKLYYNINYIEQI	Allele 31
			KITSSLSNNYNSNNYNKYNYNNSKKLYYNINYIEQI	Allele 2
L2FW	L2FQ	L2Q	KIISSLSNKTIHNNNNYNNNNYNNYKKLYYNINYIEQI	Allele 14
			KIISSLSNNTIHNNNNYKYNYNNNYNNYNNKKLYYNINYIEQI	Allele 35
L2GW	L2GQ	L2Q	KIISSLSNKTIHNNNNYKYNYNNKYNYNNNNYNKKLYYKNYIINIEQI	Allele 3
			KITSSLSNNYNSNNYNKYNYNNSKKLYYNINYIEQI	Allele 2
L2HW	L2HQ	L2Q	KIISSLSNKTIHNNNKYNYNKYNYNNNNYNNYKKLYYNINYIEQI	Allele 4
			KIISSLSNKTIHNNNNYKYNYNNKYNYNNNNYNKKLYYKNYIINIEQI	Allele 3
L2IW	L2IQ	L2Q	KITSSLSNNYNSNNYNKYNYNNSKKLYYNINYIEQI	Allele 2
			KIISSLSNKTIHNNNNYKNYNYKKLYYNIINIEQI	Allele 1
L3AW	L3AQ	L3Q	KIISSLSNKTIHNNNNYNNNNYNNYKKLYYNINYIEQI	Allele 14
			KIISSLSNNTIHNNNYKYNYNNNYNNYKKLYYNINYIEQI	Allele 25
L3BW	L3BQ	L3Q	KIISSLSNNTIHNNNYKYNYNNNNYNNNNYNKKLYYNIINIEQI	Allele 32
			KIISSLSNKTIHNNNKYNYNKYNYNNNNYNNYKKLYYNINYIEQI	Allele 4
L4AW	L4AQ	L4Q	KIISSLSNNYKYSNYNNYNNNNNYNNNYSKKLYYNIINIEQI	Allele 6
			KIISSLSNNYKYSNYNNYNNNYNNYKKLYKNYIINIEQI	Allele 27
L4BW	L4BQ	L4Q	KITSSLSNSCNYSNNYNNNYNNTKKLYYNINYIEQI	Allele 30
			KIISSLSNKTIHNNNNYKYNYNNNNNNYKNYNNYKKLYYNINYIEQI	Allele 17
L5AW	L5AQ	L5Q	KIISSLSNKTIHNNNKYNYNKYNYNNNNYNNYKKLYYNINYIEQI	Allele 4
			KIISSLSNNYISNISNYNNNNNSKKLYYNINYIEQI	Allele 16
L5BW	L5BQ	L5Q	KIISSLSNNYNYSNYNNNNNNYNNYKQLCYNINYIEQI	Allele 5
			KIISSLSNKTIHNNNNYKYNYNNNNNNYKNYNNYKKLYYNINYIEQI	Allele 17
L5CW	L5CQ	L5Q	KIISSLSNKTIHNNNNYKYNYNNNNYNNNNYNNNNYKKLQYYNINYIEQI	Allele 15
			KIISSLSNKTIHNNNNYNNNNYNNYKKLYYNIINIEQI	Allele 19
L5DW	L5DQ	L5Q	KIISSLSNNYNYSNYNNNNNNYNNYKQLCYNINYIEQI	Allele 5
			KIISSLSNKTIHNNNNYKNYNYKKLYYNIINIEQI	Allele 1
L5EW	L5EQ	L5Q	KIISSLSNNTIHNNNNYNKKLYYNIINIEQI	Allele 29
			KIISSLSNKTIHNNNNYKYNYNNNNYKNYNNYKKLYYNINYIEQI	Allele 18
L5FW	L5FQ	L5Q	KIISSLSNNYNYSNYNNYNNNNYNNYKKLYYNINYIEQI	Allele 23
			KIISSLSNNYKYSNYNNYNNNYNNYNNNYKKLYYNINYIEQI	Allele 34
L5GW	L5GQ	L5Q	KIISSLSNNYISNISNYNNNNNSKKLYYNINYIEQI	Allele 16
			KIISSLSNKTIHNNNKYNYNKYNYNNNNYNNYKKLYYNINYIEQI	Allele 4
L6AW	L6AQ	L6Q	KIISSLSNNYNYNNCNYKHNKLYYNIINIEQI	Allele 24
			KIISSLSNNYNYNNNNYNNYNNNYNNNYNKKLYYNIINIEQI	Allele 22
L7AW	L7AQ	L7Q	KIISSLSNKTIHNNNNYKKLYYNINYIEQI	Allele 9
			KIISSLSNKTIHNNNNYKNYNYKKLYYNIINIEQI	Allele 1
L7BW	L7BQ	L7Q	KIISSLSNKTIHNNNNYKYNYNNKYNYNNNNYNNNNYNKKLYYKNYIINIEQI	Allele 20
			KIISSLSKNTIHNNNYKYNYNNNNNYNNNYKKLQYYNINYIEQI	Allele 12
L7CW	L7CQ	L7Q	KIISSLSNNYNSNSLLSYNNYNNNNNYNKLYYNINYIEQI	Allele 47
			KIISSLSKNTIHNNNYKYNYNNNNNYNNNYKKLQYYNINYIEQI	Allele 12
L7DW	L7DQ	L7Q	KIISSLSKNTIHNNNYKYNYNNNNNYNNNYKKLQYYNINYIEQI	Allele 12
			KIISSLSNNYNYSNYNNNNNNYNNYKQLCYNINYIEQI	Allele 5
L7EW	L7EQ	L7Q	KIISSLSNNTIHNNNYKYNYNNNYNNYNNYKKLYYNINYIEQI	Allele 7
			KIISSLSNKTIHNNNNYKNYNYKKLYYNIINIEQI	Allele 1
L7FW	L7FQ	L7Q	KIISSLSNKTIHNNNNYKNYNYKKLYYNIINIEQI	Allele 1
			KIISSLSNKTIHNNNNYKNYNNYNNNNYKNYNNYKKLYYNIINIEQI	Allele 11
L8AW	L8AQ	L8Q	KIISSLSNNTIHNNNYKYNYNNNYNNYNNYKKLYYNINYIEQI	Allele 7
			KIISSLSNNTIHNNNYKYNYNNNNYNKKLYYNIINIEQI	Allele 38
L8BW	L8BQ	L8Q	KIISSLSNNYNYNNNYNNYNNNYNKKLYYNINYIEQI	Allele 39
			KIISSLSNKTIHNNNKYNYNKYNYNNNNYNNYKKLYYNINYIEQI	Allele 4
L8CW	L8CQ	L8Q	KIISSLSNHYNYNNNKYNNYNNDYKKLYYNINYIEQI	Allele 41
			KIISSLSNNTIHNNNYKYNYNNNYNNYNNYKKLYYNINYIEQI	Allele 7
L8DW	L8DQ	L8Q	KIISSLSNNYKYSNYNNYNNYNKKLYYKNYIINIEQI	Allele 8
			KIISSLSNKTIHNNNNYKNYNYKKLYYNIINIEQI	Allele 1
L8EW	L8EQ	L8Q	KIISSLSNKTIHNNNNYKNYNYKKLYYNIINIEQI	Allele 1
			KIISSLSNNYKYSNYNNYNNYNKKLYYKNYIINIEQI	Allele 8
L9AW	L9AQ	L9Q	KITSSLSNNYNSNNYNKYNYNNSKKLYYNINYIEQI	Allele 2
			KIISSLSNKTIHNNNNYKYNYNNNNYNNNNYNNNYNNNCKKLYYNIINIEQI	Allele 10
L9BW	L9BQ	L9Q	KIISSLSNKTIHNNNNYKYNYNNKYNYNNNNYNNNNYNKKLYYKNYIINIEQI	Allele 20
			KIISSLSNKTIHNNNNYKNYNNYNNNNYKNYNNYKKLYYNIINIEQI	Allele 11
L10AW	L10AQ	L10Q	KIISSLSNNYSYNNYNNNNYNKKLYYNINYIEQI	Allele 44
			KIISSLSNNYNYNNYNNNYKPLYYNINYIEQI	Allele 21
L10BW	L10BQ	L10Q	KITSSLSNNYNSNNYNKYNYNNSKKLYYNINYIEQI	Allele 2
			KIISSLSNNYNYNNYNNNYKPLYYNINYIEQI	Allele 21
L10CW	L10CQ	L10Q	KIISSLSNKTIHNNNNYNNNNYNNYKKLYYNIINIEQI	Allele 19
			KIISSLSNNYKYSNYNNYNNNNNYNNNYSKKLYYNIINIEQI	Allele 6
L11AW	L11AQ	L11Q	KIISSLSNNTIHNNNYKYNYNNNNYNNNYNKKLYYKNYIINIEQI	Allele 36
			KIISSLSNNYKYSNYNNYNNNYNNYNNNYNNNYKKLYYNINYIEQI	Allele 37
L11BW	L11BQ	L11Q	KIISSLSNKTIHNNNNYKYNYNNKYNYNNNNYNKKLYYKNYIINIEQI	Allele 3
			KIISSLSNKTIHNNNNYNNNNNNYNNYNNYKKLYYNVINIEQI	Allele 43
L11CW	L11CQ	L11Q	KIISSLSNNRNSNNYNNYNYKKLYYNINYIEQI	Allele 45
			KIISSLSNNTIHNNNNYKYNYNNNYNNYNNYNNKKLYYNINYIEQI	Allele 46
L11DW	L11DQ	L11Q	KIISSLSNKTIHNNNNYKNYNYKKLYYNIINIEQI	Allele 1
			KIISSLSNKTIHNNNNYKYNYNNKYNYNNNNYNKKLYYKNYIINIEQI	Allele 3
L11EW	L11EQ	L11Q	KIISSLSNNTIHNNNYKYNYNNNYNNYNNYKKLYYNINYIEQI	Allele 7
			KIISSLSNKTIHNNNNYKYNYNNNNYNNNNYNNNYNNNCKKLYYNIINIEQI	Allele 10
L12AW	L12AQ	L12Q	KIISSLSNKTIHNNNNYKYNYNNNYNNNHYNNNYKKLQYYNIINIEQI	Allele 40
			KIISSLSNNYKYSNYNNYNNNNNYNNNYSKKLYYNIINIEQI	Allele 6
L12BW	L12BQ	L12Q	KIISSLSNNYKYSNYNNYNNNNNYNNNYSKKLYYNIINIEQI	Allele 6
			KIISSLSNKTIHNNNNYKYNYNNNNYNNNNYNNYNNNCKKLYYNIINIEQI	Allele 42
L12CW	L12CQ	L12Q	KIISSLSNKTIHNNNNYKNYNNYNNNNYKNYNNYKKLYYNIINIEQI	Allele 11
			KIISSLSNNYNYSNYNNNNNNYNNYKQLCYNINYIEQI	Allele 5
L12DW	L12DQ	L12Q	KITSSLSNNYNSNNYNKYNYNNSKKLYYNINYIEQI	Allele 2
			KIISSLSNKTIHNNNNYKKLYYNINYIEQI	Allele 9
L12EW	L12EQ	L12Q	KIISSLSNKTIHNNNNYKKLYYNINYIEQI	Allele 9
			KIISSLSNNYKYSNYNNYNNNNNYNNNYSKKLYYNIINIEQI	Allele 6
L13AW	L13AQ	L13Q	KIISSLSNKTIHNNNNYKYNYNNKYNYNNNNYNKKLYYKNYIINIEQI	Allele 3
			KIISSLSNKTIHNNNNYKYNYNNNNYNNNNYNNNYNNNCKKLYYNIINIEQI	Allele 10

## Data Availability

Amino acid sequences of the *csd* alleles are given in the manuscript. Nucleotide sequences of the novel *csd* hypervariable regions described in this work are available on Genbank with the following accession number: BankIt2716350 Allele1 OR180323, BankIt2716350 Allele2 OR180324, BankIt2716350 Allele3 OR180325, BankIt2716350 Allele4 OR180326, BankIt2716350 Allele5 OR180327, BankIt2716350 Allele6 OR180328, BankIt2716350 Allele7 OR180329, BankIt2716350 Allele8 OR180330, BankIt2716350 Allele9 OR180331, BankIt2716350 Allele10 OR180332, BankIt2716350 Allele11 OR180333, BankIt2716350 Allele12 OR180334, BankIt2716350 Allele13 OR180335, BankIt2716350 Allele14 OR180336, BankIt2716350 Allele15 OR180337, BankIt2716350 Allele16 OR180338, BankIt2716350 Allele17 OR180339, BankIt2716350 Allele18 OR180340, BankIt2716350 Allele19 OR180341, BankIt2716350 Allele20 OR180342, BankIt2716350 Allele21 OR180343, BankIt2716350 Allele22 OR180344, BankIt2716350 Allele23 OR180345, BankIt2716350 Allele24 OR180346, BankIt2716350 Allele25 OR180347, BankIt2716350 Alelle26 OR180348, BankIt2716350 Allele27 OR180349, BankIt2716350 Allele28 OR180350, BankIt2716350 Allele29 OR180351, BankIt2716350 Allele30 OR180352, BankIt2716350 Allele31 OR180353, BankIt2716350 Allele32 OR180354, BankIt2716350 Allele33 OR180355, BankIt2716350 Allele34 OR180356, BankIt2716350 Allele35 OR180357, BankIt2716350 Allele36 OR180358, BankIt2716350 Allele37 OR180359, BankIt2716350 Allele38 OR180360, BankIt2716350 Allele39 OR180361, BankIt2716350 Allele40 OR180362, BankIt2716350 Allele41 OR180363, BankIt2716350 Allele42 OR180364, BankIt2716350 Allele43 OR180365, BankIt2716350 Allele44 OR180366, BankIt2716350 Allele45 OR180367, BankIt2716350 Allele46 OR180368, BankIt2716350 Allele47 OR180369.

## Supplementary Materials

The following supporting information can be downloaded at https://www.mdpi.com/article/10.3390/genes15060652/s1: Table S1: Amino acid sequences of the hypervariable region of the *csd* gene and their lengths (L = nr of aa). Amel*csd*-HVR accession numbers or Paolillo Allele numbers are reported for the alleles already reported by Bilodeau and Elsik 2021 or Paolillo et al. 2022 [19,20].
