# Supplementary material for: Variability and Number of Circulating Complementary Sex Determiner (Csd) Alleles in a Breeding Population of Italian Honeybees under Controlled Mating
Source: Genes (Basel). 2024 May 21;15(6):652. doi: 10.3390/genes15060652 (PMC11202483; doi:10.3390/genes15060652)
Supplement: Supplementary file 1 [file genes-15-00652-s001.zip › genes-3000784-supplementary.pdf]

Table S1. Amino acid sequences of the hypervariable region of the *csd* gene and their lengths (L = nr of aa). Amel*csd*-HVR accession numbers or Paolillo Allele numbers are reported for the alleles already reported by Bilodeau and Elsik 2021 or Paolillo et al. 2022 [19, 20].

ID allele	sequences	L	Amel*csd*-HVR ID/ Paolillo et al. ID	frequency
Allele 1	KIISSLSNKTIHNNNNYKNYNYKKLYYNIINIEQI	35	Amel*csd*-HVR115	8%
Allele 2	KITSSLSNNYNSNNYNKYNYNNSKKLYYNINYIEQI	36	Amel*csd*-HVR213	7%
Allele 3	KIISSLSNKTIHNNNNYKYNYNNKYNYNNNNYNKKLYYKNYIINIEQI	48	Amel*csd*-HVR70	7%
Allele 4	KIISSLSNKTIHNNNKYNYNKYNYNNNNYNNYKKLYYNINYIEQI	45	Amel*csd*-HVR107	7%
Allele 5	KIISSLSNNYNYSNYNNNNNNYNNYKQLCYNINYIEQI	38	Amel*csd*-HVR42	5%
Allele 6	KIISSLSNNYKYSNYNNYNNNNNYNNNYSKKLYYNIINIEQI	42	Amel*csd*-HVR166	5%
Allele 7	KIISSLSNNTIHNNNYKYNYNNNYNNYNNYKKLYYNINYIEQI	43	Paolillo Allele 4	4%
Allele 8	KIISSLSNNYKYSNYNNYNNYNKKLYYKNYIINIEQI	37	Amel*csd*-HVR21	3%
Allele 9	KIISSLSNKTIHNNNNYKKLYYNINYIEQI	30	Amel*csd*-HVR111	3%
Allele 10	KIISSLSNKTIHNNNNYKYNYNNNNYNNNNYNNNYNNNCKKLYYNIINIEQI	52	Amel*csd*-HVR60	3%
Allele 11	KIISSLSNKTIHNNNNYKNYNNYNNNNYKNYNNYKKLYYNIINIEQI	47	Amel*csd*-HVR114	3%
Allele 12	KIISSLSKNTIHNNNYKYNYNNNNNYNNNYKKLQYYNINYIEQI	44	Amel*csd*-HVR99	3%
Allele 13	KIISSLSNKTIHNNNNYKYNYNNNNYNNNYNNYKKLYYNINYIEQI	46	Amel*csd*-HVR23	2%
Allele 14	KIISSLSNKTIHNNNNYNNNNYNNYKKLYYNINYIEQI	38	Paolillo Allele 16	2%
Allele 15	KIISSLSNKTIHNNNNYKYNYNNNNYNNNNYNNNNYKKLQYYNINYIEQI	50	Amel*csd*-HVR124	2%
Allele 16	KIISSLSNNYISNISNYNNNNNSKKLYYNINYIEQI	36	Amel*csd*-HVR38	2%
Allele 17	KIISSLSNKTIHNNNNYKYNYNNNNNNYKNYNNYKKLYYNINYIEQI	47	Amel*csd*-HVR86	2%
Allele 18	KIISSLSNKTIHNNNNYKYNYNNNNYKNYNNYKKLYYNINYIEQI	45	Amel*csd*-HVR120	2%
Allele 19	KIISSLSNKTIHNNNNYNNNNYNNYKKLYYNIINIEQI	38	Amel*csd*-HVR134	2%
Allele 20	KIISSLSNKTIHNNNNYKYNYNNKYNYNNNNYNNNNYNKKLYYKNYIINIEQI	53	Amel*csd*-HVR20	2%
Allele 21	KIISSLSNNYNYNNYNNNYKPLYYNINYIEQI	32	Amel*csd*-HVR12	2%
Allele 22	KIISSLSNNYNYNNNNYNNYNNNYNNNYNKKLYYNIINIEQI	42	Amel*csd*-HVR43	1%
Allele 23	KIISSLSNNYNYSNYNNYNNNNYNNYKKLYYNINYIEQI	39	Amel*csd*-HVR195	1%
Allele 24	KIISSLSNNYNYNNCNYKHNKLYYNIINIEQI	32	Amel*csd*-HVR34	1%
Allele 25	KIISSLSNNTIHNNNYKYNYNNNYNNYKKLYYNINYIEQI	40	Amel*csd*-HVR158	1%
Allele 26	KIISSLSNNYNYSNYNNYNNNNNYNNYKKLYYNINYIEQI	40	Amel*csd*-HVR13	1%
Allele 27	KIISSLSNNYKYSNYNNYNNNYNNYKKLYKNYIINIEQI	39	Amel*csd*-HVR52	1%
Allele 28	KIISSLSNNYKYSNYNNYNNNYNNNYNNNYNNNYKKLYKNYIINIEQI	48	Amel*csd*-HVR39	1%
Allele 29	KIISSLSNNTIHNNNNNYNKKLYYNIINIEQI	31	Amel*csd*-HVR19	1%
Allele 30	KITSSLSNSCNYSNNYNNNYNNTKKLYYNINYIEQI	36	Amel*csd*-HVR25	1%
Allele 31	QIISSLSNNYNYNYNKHNNKLYYNINYIEQI	31	Amel*csd*-HVR82	1%
Allele 32	KIISSLSNNTIHNNNYKYNYNNNNYNNNNYNKKLYYNIINIEQI	43	Amel*csd*-HVR31	1%
Allele 33	KIISSLSNKTIHNNNNYKNYNNYKKLYYNINYIEQI	36	Amel*csd*-HVR3	1%
Allele 34	KIISSLSNNYKYSNYNNYNNNYNNYNNNYKKLYYNINYIEQI	42	Amel*csd*-HVR36	1%
Allele 35	KIISSLSNNTIHNNNNYKYNYNNNYNNYNNKKLYYNINYIEQI	43	-	1%
Allele 36	KIISSLSNNTIHNNNYKYNYNNNNYNNNYNKKLYYKNYIINIEQI	45	Amel*csd*-HVR157	1%
Allele 37	KIISSLSNNYKYSNYNNYNNNYNNYNNNYNNNYKKLYYNINYIEQI	46	Amel*csd*-HVR170	1%
Allele 38	KIISSLSNNTIHNNNYKYNYNNNNYNKKLYYNIINIEQI	39	Amel*csd*-HVR155	1%
Allele 39	KIISSLSNNYNYNNNYNNYNNNYNKKLYYNINYIEQI	37	-	1%
Allele 40	KIISSLSNKTIHNNNNYKYNYNNNYNNNHYNNNYKKLQYYNIINIEQI	48	Amel*csd*-HVR68	1%
Allele 41	KIISSLSNHYNYNNNKYNNYNNDYKKLYYNINYIEQI	37	Amel*csd*-HVR51	1%
Allele 42	KIISSLSNKTIHNNNNYKYNYNNNNYNNNNYNNYNNNCKKLYYNIINIEQI	51	-	1%
Allele 43	KIISSLSNKTIHNNNNYNNNNNNYNNYNNYKKLYYNVINIEQI	43	Amel*csd*-HVR54	1%
Allele 44	KIISSLSNNYSYNNYNNNNYNKKLYYNINYIEQI	34	Amel*csd*-HVR65	1%

Allele 45	KIISSLSNNRNSNNYNNYNYKKLYYNINYIEQI	33	Amel*csd*-HVR64	1%
Allele 46	KIISSLSNNTIHNNNNYKYNYNNNYNNYNNYNNKKLYYNINYIEQI	46	Amel*csd*-HVR28	1%
Allele 47	KIISSLSNNYNSNSLLSYNNYNNNNNYNKLYYNINYIEQI	40	Amel*csd*-HVR175	1%
